# Supplementary material for: Netcombin: An algorithm for constructing optimal phylogenetic network from rooted triplets
Source: PLoS One. 2020 Sep 18;15(9):e0227842. doi: 10.1371/journal.pone.0227842 (PMC7500971; doi:10.1371/journal.pone.0227842)
Supplement: S4 File — (ZIP) [file pone.0227842.s004.zip › SequenceData-Triplet/sequenceData-10-20-40-Triplet2/data-(sample 5-length 500- num seq 40- m rate 10^-7)/5 seq.pdf]

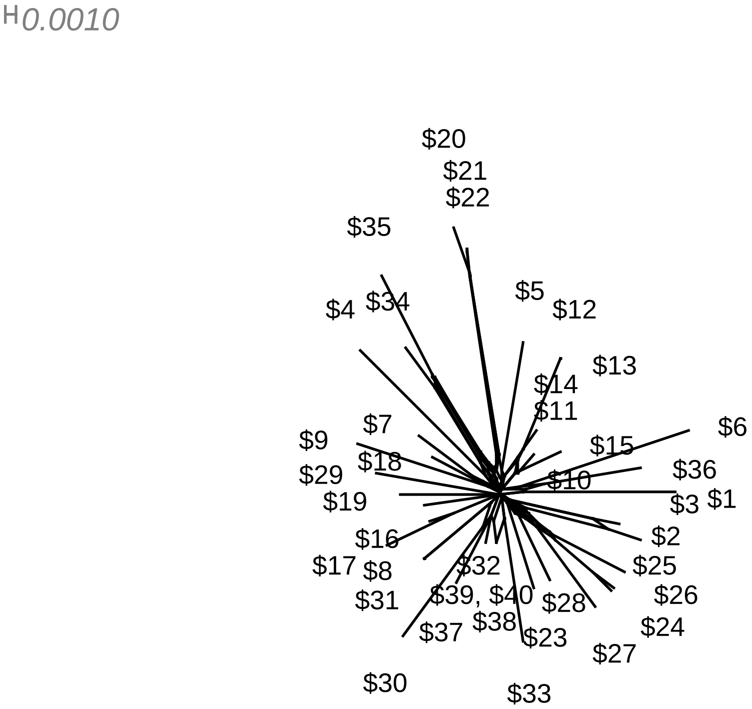
0.0010
$20
$21
$22
$35
$5
$12
$4
$34
$13
$14
$11
$7
$6
$9
$15
$18
$36
$29
$10
$19
$3
$1
$16
$2
$17
$8
$32
$25
$26
$31
$39, $40
$28
$38
$24
$37
$23
$27
$30
$33
